# LFSD: a VSLAM dataset with plant detection and tracking in lettuce farm

**DOI:** 10.3389/fpls.2023.1175743

**Published:** 2023-08-29

**Authors:** Shuo Wang, Daobilige Su, Maofeng Li, Yiyu Jiang, Lina Zhang, Hao Yan, Nan Hu, Yu Tan

**Affiliations:** ^1^ College of Engineering, China Agricultural University, Beijing, China; ^2^ Beijing Zhong Nong LV Tong Agriculture Development LTD, Beijing, China

**Keywords:** dataset, agriculture, robotics, SLAM, MOT, lettuce, detection

## 1 Introduction

The rapid development of robotics and artificial intelligence has led to increasing deployment of agricultural robots for precision agricultural applications (Chebrolu et al., 2017; Su et al., 2021; Hu et al., 2022b). Simultaneous Localization and Mapping (SLAM) is a critical skills for robots, which builds the environmental map around the robot and localizes the robot on the map at the same time (Cadena et al., 2016). SLAM is also a prerequisite of many other tasks for robots, such as autonomous navigation (Ponnambalam et al., 2020) and path planning (Bonny and Kashkash, 2022). Various modalities of sensors are used to realize SLAM, so as to realize full automation of agricultural robots (Gupta and Fernando, 2022; Tourani et al., 2022). Among them, Visual Simultaneous Localization and Mapping (VSLAM) has gained tremendous attention due to the wide availability of camera and its cost effective nature (Matsuki et al., 2018; Campos et al., 2020). Many datasets have been proposed for VSLAM, such as KITTI dataset (Geiger et al., 2013) and TUM dataset (Schubert et al., 2018). These benchmark datasets are of key importance to make a fair comparison and validation of different VSLAM methods. Therefore, construction of a benchmark dataset captured in agricultural field for VSLAM is important for design and evaluation of VSLAM methods that are suitable for agricultural robots.

In recent years, the number of publicly available datasets in the field of robotic application in agricultural has gradually increased, attracting surging attention from researchers. Currently, agricultural datasets mostly focus on fruit detection (Gené-Mola et al., 2019), weed detection (Dos et al., 2017; Olsen et al., 2018) and obstacle avoidance applications (Pezzementi et al., 2018). Only few datasets are available for localization and navigation, and further processing of the data is lacking. Hansen et al. (2017) collected data from stereo camera, thermal camera, LIDAR, Inertial Measurement Unit (IMU), and GNSS in dynamic scenes, and added object labels and geographic coordinates to all static and moving obstacles. The dataset is primarily used for localization and obstacle detection of robots in agricultural field. Zujevs et al. (2021) proposed the first event-based vision dataset, recording data sequences in 12 different scenes in autumn, aiming to cover visual navigation tasks in different types of agricultural environments. The dataset is specifically designed for a special type of vision sensor, *i.e.* event camera. Chebrolu et al. (2017) presented a multi-sensor dataset for plant segmentation, localization and mapping in a sugar beet farm. The dataset consists of data captured by RGB-NIR camera, Kinect RGB and depth camera and RTK-GPS sensor, and is recorded continuously for three months. The RGB-NIR camera data is semantically annotated for pixel-wise classification and segmentation of sugar beet and weed. Pire et al. (2018) proposed a number of visually challenging soybean field scenarios captured by ZED stereo camera, including sunlight reflections, irregular terrain, and highly repetitive texture which is particularly challenging for loop closure. In addition, information such as IMU and wheel odometers were recorded for the evaluation of SLAM algorithms by the fusion of multiple sensors. The captured RTK-GPS serves as ground truth for robot trajectories. Aiming at the dynamic characteristics and change of plant features in agricultural environments, Dong et al. (2016) performed continuous recordings of a peanut field. A data association algorithm is designed to solve the problem of large appearance change caused by different time points and different angles. Lu and Young (2020) and Wang et al. (2022) provided a review on agricultural datasets for robotics.

Regarding VSLAM of agricultural robots in farms or fruit orchards, the situation is usually more challenging than the most general case, due to the semi-structured environment of farms and fruit orchards. Although the plants and trees are often planted in structured rows, VSLAM is still a challenging problem, because of the repetitive visual pattern which is observed by robots when driving along these rows. The repetitive visual pattern can severely damage the performance of VSLAM by introducing incorrect matching of visual feature points and incorrect loop closure. Classical VSLAM frameworks often use direct or semi-direct methods such as LSD-SLAM (Engel et al., 2014) and DSO (Matsuki et al., 2018), and indirect methods such as PTAM (Klein and Murray, 2007) and ORB-SLAM3 (Campos et al., 2020), to optimize camera poses and build the environmental map. Due to the semi-structured nature of the farms and fruit orchards, these conventional VSLAM methods often fail or perform poorly when being used in agricultural dataset. To effectively resolve the challenging semi-structured environment, it is important for robots to fully exploit objects and semantic information in their surrounding environment (Wang et al., 2020). Recent works in VSLAM show that adding object (Yang and Scherer, 2018) and semantic level information (Wen et al., 2021) to conventional visual feature points yields promising results. Among them, object SLAM is a typical application of semantic SLAM, which aims to estimate more robust and accurate camera poses by leveraging the semantic information of in-frame objects (Wu et al., 2020).

In this paper, Lettuce Farm SLAM Dataset (LFSD), a VSLAM dataset based on RGB and depth images captured by VegeBot (Hu et al., 2022a) in a lettuce farm, is presented. The dataset consists of RGB and depth images, IMU, and RTK-GPS sensor data. Detection and tracking of lettuce plants on images are annotated with the standard Multiple Object Tracking (MOT) format (Zhang et al., 2020). It aims to accelerate the development of algorithms for localization and mapping in the agricultural field, and crop detection and tracking. Our data and related supporting documents is publicly released at https://ieeedataport.org/documents/lfsd-dataset and https://github.com/wangshuo9707/LFSD-Dataset. Supplementary python scripts for converting raw data files (text and JPG image files) to ROS bag files for ROS1 and ROS2 systems, as well as converting MOT annotation files to target detection annotation files, are provided for the convenience of users.

**Figure 1:**
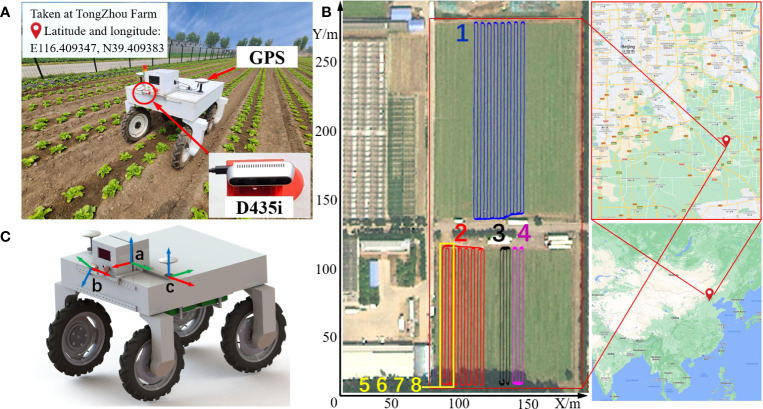
Details of data acquisition. **(A)** Robot and installed sensors. **(B)** Geological location of the farm and GPS trajectories of the eight sequences of the dataset. **(C)** Illustration of the coordinate frames. The a is *base*_*link*, the b is *D*435*i*_*link*, and the c is *GPS*_*link*. The *x*-axis is colored red, the *y*-axis is green, and the *z*-axis is blue.

The major contributions of the proposed dataset are summarized as follows:

1. Eight closed-loop sequences recorded in a lettuce farm by the VegeBot robot are provided, as shown in **Figure 1A**. The dataset is approximately 67 G, including RGB images, depth images and IMU information recorded by the Intel RealSense D435i sensor installed in front of the robot slightly facing downward. The MOT (Zhang et al., 2020) format is used to annotate part of the RGB and depth images, which is used to carry out research on detection and tracking of lettuce plants. RTK-GPS data is presented for performance evaluation of VSLAM algorithms. We tested three open source VSLAM algorithms with the proposed dataset, and report benchmark results for comparison purpose.
2. Compared to existing datasets for robotic localization and mapping in agricultural field, the proposed dataset provides plant detection and tracking annotation, which makes it possible for object level VSLAM. In addition, recording of closed-loop data in different growth stages of lettuces from the same area is helpful for designing a spatio-temporal model of a dynamic scene. Specifically, the dataset provided by Pire et al. (2018) does not contain any plant detection information. Though the dataset provided by Chebrolu et al. (2017) provided semantic labels for RGB-NIR image pairs, there is no straightforward object labels for each individual plants and correlations between plants in consecutive images. Therefore, it can not be directly used for object level VSLAM. Even if the RGB-NIR data can be post-processed to extract all objects and their correspondences for object level VSLAM, the resulting localization lacks scale information since the RGB-NIR camera is essentially a single pinhole camera. In comparison, our method directly provides plant detection and tracking information for RGB and depth image pairs, which is suitable to evaluate object level VSLAM without lack of scale information.

## 2 Materials and methods

### 2.1 Data acquisition

The dataset was collected in the spring of 2022 and 2023, respectively, at a lettuce farm in Tongzhou district, Beijing, China. The lettuce is planted in a ridge transplanting mode, and two rows are planted on each ridge. The distance between rows and plants is about 35cm. At the time of data collection, the lettuce was in the rosette stage, and there is no obvious overlap between plants. The VegeBot robot used to collect data is developed by China Agricultural University. It is powered by 8 servo motors for four-wheel independent drive and steering, so that it can drive in various farms with great flexibility as shown in **Figure 1A**. The VegeBot is equipped with a RTK-GPS sensor for GNSS-based global positioning and a forward-tilted Intel RealSense D435i depth camera with an IMU sensor. In order to ensure the recording quality of the dataset, the robot is remotely controlled following the middle of the plant rows as much as possible with a speed of about 0.6m/s. When the robot is driving straight along the lettuce ridge, it adopts the front-wheel Ackerman steering method to ensure smooth progress. When turning at the end of the ridge, it adopts synchronous four-wheel steering to provide the smallest turning radius.

The Intel RealSense D435i depth camera is approximately 1.1m high from the ground and tilted slightly downward with an angle of 40^°^ between horizontal line. For Intel RealSense D435i camera, its RGB Field of View (FOV) is 69^°^ ×42^°^, the maximum resolution is 1920×1080, and the frame rate is 30 Frames Per Second (FPS). The FOV of depth image can reach 87^°^ ×58^°^, the maximum resolution is 1280×720, the maximum frame rate is 90 FPS, and the depth accuracy is< 2%. It provides a wide field of view in a global shutter mode, so it can cover a wider area and has high adaptability to low-light environments. An IMU is also available to measure acceleration and rotation rate with 6 Degree Of Freedom (DOF). During the data collection, it streams RGB images with a size of 1280×720, and aligned depth images at 10 FPS. It streams IMU information at 200 HZ. The RTK-GPS sensor based on GNSS global positioning receives the satellite signal and the differential signal of the base station, and performs RTK calculation internally. Finally, the longitude and latitude position information with centimeter-level precision and direction information are released at 1HZ. All sensor data is recorded by the on-board computer with Intel i5-9400 CPU, NVIDIA RTX1650 GPU, 8 GB DDR4 RAM, 1T hard disk, and operating system of Ubuntu 18.04. Sensor data is recorded with their Robot Operating System (ROS) drivers in terms of asynchronous ROS topics. Timestamp is recorded for each piece of information, which is used for synchronizing data from multiple sensors.

In total, the robot drives eight closed loop trajectories. With each trajectory, the robot starts from the first ridge, continuously drives through the lettuce field with multiple ridges, finally returns to the first ridge, and drives for a certain distance, as shown in **Figure 1B**. Sequences 1 to 4 are closed-loop recordings from four different areas recorded in 2022. Among them, Sequences 1 and 2 cover large area of the farm. Due to the regular weed removal in the field of sequence 1, the density of weed is low throughout the planting period. In comparison, in order to enhance the richness and complexity of the dataset, the areas where sequences 2, 3, and 4 are located have not been manually cleared of weeds, so the density of weed is relatively high. Sequences 5 to 8 are closed-loop recordings of the same area captured in 2023. In these four sequences, the weed density is comparatively low. The resolution of RGB and depth images recorded by the D435i sensor is 640×480. The tilt angle between the sensor and the horizontal line is set to 45 degree to obtain a wider field of view.

### 2.2 Dataset

#### 2.2.1 Extrinsics between different coordinates

To facilitate the data fusion of different sensors, we provide the 3D coordinate transformations between the *base*_*link* of the robot and all other sensors. Among them, the RTK-GPS has two satellite signal receiving antennas, which run in master slave mode. The master antenna on the left side serves as the base point of positioning, which is denoted as *GPS*_*link*. The slave antenna on the right side assists the master antenna in positioning by providing the orientation of *yaw* and *pitch* angles. The RTK-GPS sensor does not provide the *roll* angle. The robot *base*_*link* is located in the middle of two GPS antennas over its Y axis direction, as shown in **Figure 1C**. The coordinate transformations of other sensors relative to the *base*_*link* are shown in **Table 1**, where the translation is given by *x*, *y* and *z*, and the rotation is given by the quaternion.

**Table 1:** Rigid transformation from different sensors to the robot *base*_*link*.

Frame	*x*(m)	*y*(m)	*z*(m)	*qx*	*qy*	*qz*	*qw*
*base*_*link*	0	0	0	0	0	0	1
*GPS*_*link*	0	-0.491	0	0	0	-0.707	0.707
*D435i*_*link1*	-0.383	0	-0.034	-0.242	-0.242	-0.665	0.665
*D435i*_*link2*	-0.383	0	-0.034	-0.271	-0.271	-0.653	0.653

^1^Extrinsic transformation of D435i sensor relative to base_link in sequences 1, 2, 3, and 4.

^2^Extrinsic transformation of D435i sensor relative to base_link in sequences 5, 6, 7, and 8.

#### 2.2.2 Dataset structure and image annotation

**Figure 2:**
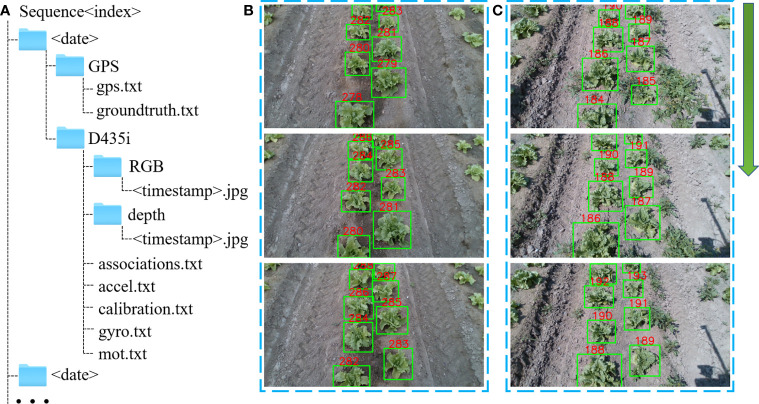
Dataset format and image annotation. **(A)** File structure for dataset. The the term<index> identifies each piece of data, the term<date> refers to the day of the data acquisition, and the term<timestamp>is the timestamp recorded by ROS. **(B)** Example of annotated plants in the low weed density scene of sequence 1. **(C)** Example of annotated plants in the high weed density scene of sequence 2.

There are eight sequences in the dataset, corresponding to the eight trajectories in **Figure 1B**. In order to prevent data loss during recording, it is saved every 5 to 8 minutes, so each sequence is split into multiple files in chronological order. For the convenience of users who are not familiar with ROS, the dataset originally recorded with ROS drivers is converted into image and text data files. Each sequence is subdivided into data folders for two sensors, GPS and D435i, as shown in **Figure 2A**.

##### 2.2.2.1 RTK-GPS

The gps.txt file in the GPS folder contains original GPS data, including longitude, latitude and altitude positional information as well as three-axis attitude angle information, with its format detailed as follows,


(1) {GPS<timestamp>lat, long, altitude, yaw, pitch, roll}

where *GPS< timestamp >* is the ROS timestamp. To estimate the accuracy of the visual odometry and VSLAM algorithms, The latitude and longitude coordinates (*lat*, *lon*, *altitude*) are converted into Cartesian coordinates (*x*, *y*, *z*). In the converted Cartesian coordinates, the robot initial position is

set to be the origin of the coordinate system. The orientation of the robot is expressed in quaternion. Using the extrinsic transformation between *base*_*link* and the *GPS*_*link*, the ground truth *base*_*link* trajectories are also provided. It is saved in the groundtruth.txt file, with its format detailed as follows,


(2) {GPS<timestamp>x y z qx qv qz qw},

where *x*, *y*, *z* are used to indicate the position of the robot, and *qx*, *qy*, *qz*, *qw* are quaternions used to indicate the rotation of the robot.

##### 2.2.2.2 D435i

The D435i folder contains both RGB and depth images, and uses ROS timestamps as names of the images. As there is a short interval between the timestamp published by the two topics, it is necessary to associate and synchronize the RGB and depth images. It is provided in the associations.txt file, whose format is as follows,


(3) {RGB<timestamp>, depth<timestamp>},

where *RGB< timestamp >* and *depth< timestamp >* are the corresponding image names in the

RGB folder and the depth folder, respectively. calibration.txt contains the camera intrinsic parameters, which are obtained based on the calibration method of Zhang (1999). This file consists of the camera’s intrinsic parameters *f_x_*, *f_y_*, *c_x_*, *c_y_*, the radial distortion coefficients *k*_1_, *k*_2_, *k*_3_, and the tangential distortion coefficients *p*_1_, *p*_2_. accel.txt and gyro.txt are accelerometer and gyroscope data, respectively. mot.txt contains annotated lettuce detection and tracking information expressed in the popular MOT format. In addition to detection, tracking of lettuce plants offers correlation between object detection results between consecutive images, as shown in **Figures 2B, C**, which helps to identify the same landmarks for object level SLAM methods. This is especially convenient for researchers to design object level SLAM algorithms using lettuce plants as landmarks.

The DarkLabel tool is used to label images, and the label format is:


(4) MOT_label={RGB<timestamp>,id,x,y,w,h,1,0,1},

where *RGB< timestamp >* is the name of the RGB image, *id* is the ID number of individual lettuce. *x* and *y* are the coordinates of the upper left corner of the label box, and *w* and *h* are the width and height of the label box. The last three the numbers 1,0,1 are not used in this dataset. All images of sequence 3 are annotated, while images of only one ridge of sequences 1, 2, and 4 are annotated. The detailed information is summarized in **Table 2**.

**Table 2:** Summary of the eight sequences of the proposed dataset.

Sequence	1	2	3	4	5	6	7	8
Sensors	D435i GPS							
Resolution	1280×720				640×480			
Number of images (Frame)	44018	28134	7120	7498	9555	9709	9247	10275
Number of labeled images	2437	2084	7120	1768	/	/	/	/
Tracks (Track)	889	538	2468	511	/	/	/	/
Boxes (Box)	19846	13585	51166	11138	/	/	/	/
Ridges/Labeled ridges	16/1	14/1	4/4	4/1	4/0	4/0	4/0	4/0
Weed density	Less	Normal	Many	Many	Less	Less	Normal	Normal
Loop closure	Strict	Strict	Strict	Strict	Strict	Strict	Strict	Strict
The weather	Cloudy	Sunny	Sunny	Sunny	Cloudy	Cloudy	Sunny	Sunny
Light intensity	Weak	Strong	Strong	Normal	Weak	Normal	Normal	Strong
Dynamic environment	No	No	No	No	No	No	No	No
Occlusion condition	No	No	No	No	No	No	No	No
Acquisition time	2022.4.26 P.M	2022.5.2 P.M	2022.5.2 P.M	2022.5.5 P.M	2023.4.17 P.M	2023.4.22 A.M	2023.4.26 A.M	2023.5.3 A.M

The details of data from each sensor are summarized as follows.

### 2.3 Evaluation of MOT and VSLAM algorithms with the proposed dataset

Extensive evaluation of popular VSLAM algorithms, as well as MOT methods, are conducted with the proposed dataset. All methods run on a computer with I9 10850K CPU, NVIDIA RTX 3070 GPU, 32G DDR4 RAM, and the operating system of Ubuntu 18.04.

Firstly, three popular MOT algorithms, which are namely FairMOT (Zhang et al., 2020), ByteTrack+NSA kalman filter (Du et al., 2021) and YOLOV5+SORT (Bewley et al., 2016), are tested with 1-4 sequences of the dataset. The results are shown in **Table 3**, and the ByteTrack+NSA kalman filter performs the best among all. The provided benchmark results are helpful to researchers in developing new MOT methods, and comparing their performance with existing methods based on the proposed dataset.

**Table 3:** Performance of three MOT methods with the proposed Dataset.

Sequence	Method	HOTA(%)↑	DetA(%)↑	AssA(%)↑	DetRe(%)↑	DetPr(%)↑	AssRe(%)↑	AssPr(%)↑
1	FairMOT	39.17	56.11	28.09	60.28	79.43	31.21	47.68
	ByteTrack+NSA kalman filter	**76.74**	**75.79**	**77.69**	**76.20**	**99.16**	**77.96**	**99.40**
	YOLOV5+SORT	65.79	63.86	69.66	67.18	81.98	73.49	87.86
2	FairMOT	50.03	54.08	51.06	58.58	74.97	54.51	77.05
	ByteTrack+NSA kalman filter	**78.87**	**77.64**	**80.15**	**78.84**	**97.67**	**80.75**	**98.71**
	YOLOV5+SORT	67.54	65.89	70.31	70.23	81.51	74.46	86.59
3	FairMOT	51.85	54.19	51.39	57.79	74.82	54.24	79.19
	ByteTrack+NSA kalman filter	**74.92**	**73.56**	**76.39**	**74.93**	**96.59**	**76.99**	**98.23**
	YOLOV5+SORT	64.34	62.96	67.04	66.87	79.82	71.04	84.64
4	FairMOT	44.95	52.15	39.79	55.97	75.82	42.18	65.53
	ByteTrack+NSA kalman filter	**75.35**	**73.77**	**77.03**	**74.86**	**97.31**	**77.62**	**98.33**
	YOLOV5+SORT	65.49	63.34	70.35	66.73	81.28	74.16	88.38

^a^ Symbols ↑ after the evaluation metrics indicate the value of it is the higher the better.

HOTA, DetA, AssA, DetRe, DetPr, AssRe and AssPr are comprehensive evaluation indicators for the MOT method, and their detailed explanations can be found in (Bewley et al., 2016; Zhang et al., 2020; Du et al., 2021; Hu et al., 2022a).The bold values show the best performing method.

In order to show the characteristics of the challenging semi-structured environment of the lettuce farm, we run three popular open source VSLAM algorithms, *i.e.* ORB-SLAM3 (Campos et al., 2020), DSO (Matsuki et al., 2018), and OA-SLAM (Zins et al., 2022), with the proposed dataset. Among them, OA-SLAM is an object level VSLAM. It uses the YOLOv5 deep neural net to detect objects, and uses the Hungarian algorithm to find the optimal data association (Zins et al., 2022). The performance of VSLAM algorithms is reflected by their accuracy, robustness, computational efficiency, scalability, map quality, and real-time performance. The Absolute Trajectory Error (ATE) (Sturm et al., 2012) is used to evaluate the accuracy of the VSLAM algorithms. The mean execution time to process one image is used to evaluate real-time performance of different methods. The results are shown in **Tables 4** and **5**. Among the three methods, only the ORB-SLAM3 and OA-SLAM result in complete tracking and positioning on sequence 3 and 7. All methods fail with the other six sequences, so they are only tested on the single ridge of each sequence.

**Table 4:** Performance of VSLAM algorithms with the proposed dataset in terms ATE.

Sequence	Overall length /m	Test length /m	ORB-SLAM3		OA-SLAM	DSO
			RGBD↓*^b^*	Monocular*^c^* ↓	Monocular ↓	Monocular↓
1	2565.28	154.38	6.05(3.91%)*^d^*	14.07(9.11%)	**5.46(3.53%)**	7.01(4.54%)
2	1637.35	112.80	**2.56(2.27%)**	27.49(24.37%)	7.22(6.40%)	6.79(6.02%)
3	455.37	455.37	39.99(8.78%)	/*e*	**19.22(4.22%)**	/
4	481.66	108.20	**2.18(2.01%)**	38.03(35.15%)	6.12 (5.65%)	8.31(7.68%)
5	478.22	79.31	**1.88(2.37%)**	8.17(10.30%)	3.43(4.32%)	3.22 (4.06%)
6	481.28	97.85	4.13(4.22%)	9.49(9.70%)	**0.84(0.80%)**	6.17(6.30%)
7	477.13	477.13	**11.18(2.34%)**	/	16.10(3.37%)	/
8	493.01	112.47	4.54(4.03%)	11.78(10.47%)	**1.31(1.16%)**	10.67(9.48%)

^b^Symbols ↓ after the evaluation metrics indicate the value of it is the lower the better.

^c^Only RGB images are used for monocular VSLAM. In this case, the optimum scale information is used during evaluation.

^d^The numbers in brackets represent the ratio of ATE over trajectory length (%).

^e^Failure of the method.The bold values show the best performing method.

**Table 5:** Mean execution times (ms) of VSLAM algorithms with the proposed dataset.

Sequence	Resolution	ORB-SLAM3		OA-SLAM	DSO
		RGBD↓	Monocular↓	Monocular↓	Monocular↓
1	1280×720	26.25	**26.15**	49.56	72.25
2	1280×720	**33.53**	33.64	64.51	73.16
3	1280×720	**36.62**	/	69.72	/
4	1280×720	36.03	**35.91**	70.22	77.28
5	640×480	**29.81**	32.92	35.21	37.52
6	640×480	32.21	**25.71**	38.52	41.22
7	640×480	**22.53**	/	44.54	/
8	640×480	**34.15**	34.25	49.6	43.51

The bold values show the best performing method.

The Symbols ↓ after the evaluation metrics indicate the value of it is the lower the better.

The results in **Table 4** show that in semi-structured lettuce field with repeated texture, both the ORBSLAM3 method relying on ORB features and the DSO method based on the sparse direct method are prone to failure. When the scene is large, as the driving distance increases, the visual odometry drifts quickly, and the ATE grows accordingly, which eventually leads to fail. Based on the results of ORB-SLAM3, it can be seen that using RGB and depth images yields more accurate results than relying on RGB images alone, and it is less likely to result in failure. Failure of ORB-SLAM3 happens mostly when the camera moves quickly when turning, causing blurry images, or when the soil texture is similar at headland. The DSO algorithm requires photometric calibration, and it fails where strong illumination change happens. Compared to these two methods relying on low-dimensional features, the OA-SLAM algorithm tracks lettuce plants, and exploits high level semantic information. In the monocular mode, it yields better localization accuracy than the other two algorithms. The performance of OA-SLAM degrades in scenes with high weed density,*e.g.*, sequences 4 and 7.

Therefore, it is critical to design a robust MOT method based object level VSLAM to effectively resolve the challenging situation of semi-structured environment of agricultural farms. Combined with feature points such as low-dimensional ORB features, the accuracy of camera pose estimation can be greatly improved. On the other hand, in repeated texture scenes, the success rate of loop closure detection based on lettuce plant detection can be increased compared to the conventional methods based on *e.g.* binary bag of words (Wang and Zell, 2018; Wang et al., 2020). The main goal of the proposed dataset is exactly to validate object level VSLAM based on plant detection and tracking.

The mean execution times of three VSLAM methods to process one image are summarized in **Table 5**, which are provided by their open source codes when running in offline mode. It can be seen that ORBSLAM3 yields the best real-time performance in both monocular and RGBD mode. OA-SLAM is based on ORB-SLAM2 method (Zins et al., 2022), and requires to carry out additional object detection and tracking step, which adds additional time consumption. Interestingly, DSO is reported to be generally faster than ORB-SLAM3, which disagrees with the test results of the proposed dataset. It is likely to be caused by the fact that, due to the complex texture of the soil in the challenging agricultural field, there are many pixels with significant brightness gradient change, which increases the run time for the semi-dense mapping.

## Data availability statement

The datasets presented in this study can be found in online repositories. The names of the repository/repositories and accession number(s) can be found below: https://ieee-dataport.org/documents/lfsd-dataset https://github.com/wangshuo9707/LFSD-Dataset.

## Author contributions

SW, DS, and LZ contributed to the design of the data acquisition. SW, ML, and HY collected the experimental data. SW, and YJ organized and labelled the data. SW and DS wrote the first draft of the manuscript. NH and YT wrote sections of the manuscript. All authors contributed to manuscript revision, read, and approved the submitted version.
